# Reduning alleviates sepsis-induced acute lung injury by reducing apoptosis of pulmonary microvascular endothelial cells

**DOI:** 10.3389/fimmu.2023.1196350

**Published:** 2023-07-03

**Authors:** Ziyi Wang, Zhe Guo, Xuesong Wang, Haiyan Liao, Feng Chen, Yuxin Liu, Zhong Wang

**Affiliations:** ^1^ Beijing Tsinghua Changgung Hospital, School of Clinical Medicine, Tsinghua University, Beijing, China; ^2^ Department of Cardiovascular Thoracic Surgery, Tianjin Medical University General Hospital, Tianjin, China

**Keywords:** SALI, Reduning, mTOR, Bax, apoptosis, network pharmacology

## Abstract

**Introduction:**

Sepsis-induced acute lung injury (SALI) is a critical illness with high mortality, and pulmonary microvascular endothelial cells (PMECs) barrier dysfunction is a well-documented pathogenesis of SALI. The current study aimed to investigate the underlying mechanism of Reduning (RDN) in the treatment of SALI.

**Methods:**

Network pharmacology and molecular dynamics simulation (MDS) were used to confirm the possibility of key active components of RDN combining with AKT1. Hematoxylin-eosin staining (HE) and immunohistochemistry (IHC) were used to investigate the effect of RDN *in vivo*. Immunofluorescence (IF) and co-immunoprecipitation (CoIP) were used to investigate the relationship between mammalian target of rapamycin (mTOR) and Bax in PMECs. ELISA was used to test the level of TNF-α. Flow cytometry was used to detect apoptosis. JC-1 and electron microscopy were used to evaluate mitochondrial damage. The results showed that RDN likely alleviated SALI *via* targeting AKT1.

**Results:**

*In vivo*, RDN could evidently decrease the expression levels of apoptosis-related proteins, alleviate mitochondrial damage, reduce lung tissue edema, down-regulate the level of TNF-α in the serum, and improve the mortality of sepsis in mice. *In vitro*, RDN had a significant effect on reducing the level of apoptosis-related proteins and cell apoptosis rate, while also mitigated mitochondrial damage. Furthermore, RDN could effectively lower the level of Bax in PMECs and increase the level of mTOR both *in vivo* and *in vitro*. Notably, mTOR has the ability to directly bind to Bax, and RDN can enhance this binding capability.

**Discussion:**

RDN could attenuate SALI through reducing apoptosis of PMECs, which is a promising therapeutic strategy for SALI prevention.

## 1 Introduction

Acute lung injury (ALI) and acute respiratory distress syndrome (ARDS) arising from a wide variety of lung injuries often result in fulminant respiratory failure and death. Among these, sepsis is the primary cause of in the intensive care unit (ICU). Specifically, 1 in 10 patients admitted to the ICU and 25% of hospitalized patients on mechanical ventilation are admitted to hospital because of ARDS. The in-hospital mortality of patients with severe ARDS is 46%–60% (1). Injury to pulmonary microvascular endothelial cells (PMECs), including apoptotic cell death, has been recently suggested to contribute to sepsis induced ALI (SALI). Non-cardiogenic pulmonary edema caused by increased permeability of PMECs is the key link in the pathogenesis of ARDS (2). As one of the most abundant cells in the lung, PMECs regulate the exchange of liquid and macromolecular substances between tissue fluid and blood through a monolayer arrangement on the intimal surface of pulmonary micro-vessels, constitute a semi-permeable barrier between blood and extravascular tissues, and regulate a variety of pathophysiological functions throughout the body and in the lung (3). Under the condition of sepsis, uncontrolled inflammatory response can lead to PMECs injury, and cause pathophysiological changes such as apoptosis and necrosis of PMECs, which eventually lead to pulmonary microvascular endothelial barrier dysfunction and increased permeability, and further induce pulmonary edema, refractory hypoxemia, and acute hypoxic respiratory failure (3, 4). Some scholars have confirmed that down-regulation of PMECs apoptosis and improvement of pulmonary vascular permeability can effectively inhibit sepsis-related acute lung injury (5, 6). Therefore, protecting PMECs from apoptosis is a key way to attenuate SALI.

Reduning (RDN) is a traditional Chinese medicine that is patented and extracted from *Artemisia annua* L., *Lonicera japonica* L., and *Gardenia jasminoides* J.Ellis (7). The plant name has been checked and verified using http://www.theplantlist.org on March 27, 2023. RDN is frequently used to treat acute respiratory tract infections. The three herbs used to extract RDN all possess anti-inflammatory, anti-oxidative, and immunomodulatory properties, which are potential strategies to treat lung injury (8–11). It has also been recognized as a treatment for severely and critically ill patients with coronavirus pneumonia (12–14). In the past few years, researchers have extensively studied how RDN can be used to treat sepsis and the underlying mechanisms involved. For example, Tang et al. stated that RDN can improve lung inflammation and oxidative stress by down-regulating the nuclear factor (NF)-kB pathway (15). Yang et al. found that RDN ameliorated LPS-induced ALI through decreased phosphorylation of extracellular regulated protein kinase (ERK)1/2 to suppress the formation of neutrophil extracellular traps (NETs) (16). In our previous research, we found that the activation of the phosphatidylinositol 3-kinase (PI3K)/protein kinase B (AKT) pathway by RDN could reduce the lipopolysaccharide (LPS)-induced apoptosis of human umbilical vein endothelial cells (HUVECs) (17). In this study, we further explored the underlining mechanisms by which RDN can inhibit apoptosis through the AKT pathway. To our knowledge, this is the first time the protective effect of RDN on SALI has been studied *in vivo* from the perspective of apoptosis.

## 2 Materials and methods

### 2.1 Reagents

RDN injection was acquired from Jiangsu Kanion Pharmaceutical Co, Ltd (Jiangsu, China). Sigma-Aldrich (St. Louis, USA) provided A6730 and 5% bovine serum albumin. Solarbio (Beijing, China) supplied Dulbecco’s Modified Eagle Medium (DMEM). Antibodies such as goat anti-mouse IgG secondary antibody (ab7063), goat anti-rabbit secondary antibody (ab7090), anti-p-AKT1 antibody (ab192623), anti-AKT1-antibody (ab179463), anti-anti-mTOR antibody (ab134903), anti-Bax antibody (ab182733), anti-cleaved-Caspase-3 antibody (ab2302), and anti-Caspase-3 antibody (ab184787) were obtained from Abcam (Cambridge, USA). CST (Boston, USA) provided anti-cleaved-Caspase-9 antibody (20750) and anti-Caspase-9 (9508) antibody. Biosharp (Guangzhou, China) provided fluorochrome-conjugated secondary antibody. Beyotime (Shanghai, China) provided JC-1 and 10% fetal bovine serum. Thermo Fisher Scientific (Massachusetts, USA) provided PE-anti-mouse CD86 antibody (12-0862-82) and FITC-anti-mouse CD206 antibody (MR5D3). Last, the TNF-α ELISA kit was purchased from Andygene (Beijing, China).

### 2.2 Cell culture

HUVECs were cultured under standard Conditions (37°C, 5% CO_2_) in the Public Laboratory of Tsinghua Changgung Hospital. Because we confirmed the appropriate treatment dose and time in preliminary experiments, in this study, the cells were cultured and divided as previously mentioned (17). There were four study groups: control group, RDN group (1:100 dilution), LPS (1 μg/ml) group, and LPS (1 μg/ml)+RDN (1:100 dilution) group. LPS treatment was administered for 12 h, and RDN was added 1 h in advance of LPS stimulation.

### 2.3 Animals

Wildtype (WT) male C57BL/6J mice were obtained from Beijing Hufukang Biotechnology Co., Ltd, China. Mice were aged between 6 and 8 weeks and weighted between 20 and 22 g. They were raised in a pathogen-free environment at Tsinghua University. Cecum ligation and puncture (CLP) was used to establish the SALI mice model which was already confirmed in a previous study (18). The C57BL/6J mice were divided into four groups of 12 mice each: sham, RDN, SALI, and SALI+RDN. The mice were fasted for at least 12 h and then given tribromoethanol (10 mg/kg) *via* intraperitoneal injection. The SALI and SALI+RDN groups had their cecum exposed after mid-line laparotomy and punctured twice with the 18G needle, then ligated below the ileo-cecal valve. The sham and RDN groups had their cecum exposed and sutured without causing intestinal obstruction. For the SALI+RDN group and RDN group, RDN was administered at a dose of 8 mL/kg or 16 mg/kg daily for one week prior to CLP surgery (19). Saline was injected subcutaneously in all groups at the end of the operation. After 12 h, six mice in each group were sacrificed by cervical dislocation and the right inferior lobe cortex was obtained as the experimental object. The survival conditions of each group (six mice/group) were observed for a week. All animal experiments were conducted in accordance with the Code of Ethics of Beijing Tsinghua Changgung Hospital (protocol code: NCT05095324).

### 2.4 Methods

#### 2.4.1 Bioinformatics methods

##### 2.4.1.1 Component target information query

*Artemisia annua* L., *Gardenia jasminoides* J.Ellis., and *Lonicera japonica* L. were selected as the research objects, and their corresponding active components were screened by bioinformatics method based on the Traditional Chinese Medicine Systems Pharmacology (TCMSP) analysis platform (https://tcmspw.com/tcmsp.php/). We screened for active ingredients using the PubChem database and identified 49 compounds that met the criteria of having an oral bioavailability of at least 30% and a drug likeness score of at least 0.18. We then used Swiss Target (http://www.swisstargetprediction.ch/) prediction to obtain 782 potential targets for these compounds.

##### 2.4.1.2 Search for disease targets

SALI’s target genes were obtained from three websites: the GeneCard human genome database (https://www.disgenet.org/), the NCBI database (https://www.ncbi.nlm.nih.gov/), and OMIM database (https://omim.org/). The research focused on the human body and used “sepsis-induced acute lung injury” as the search term. After merging the candidates from the dataset, the final result was 544 candidates.

##### 2.4.1.3 Venn diagram

**Figure 1:**
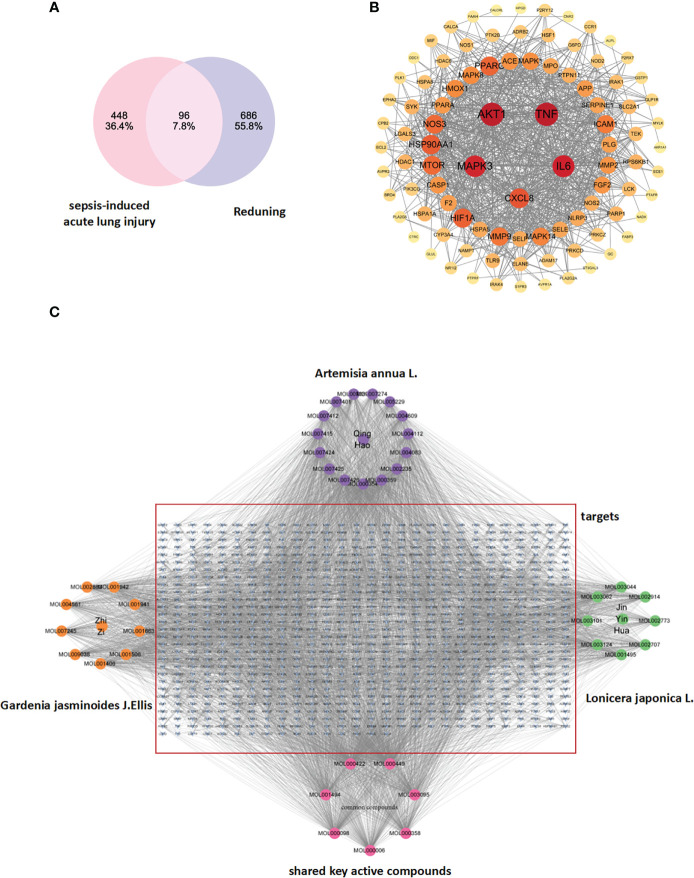
**(A)** Drug targets for RDN and sepsis targets: The identification of drug targets for both RDN and sepsis is a crucial step in developing effective treatments for these conditions. **(B)** The PPI network of 404 overlapping gene symbols provides a comprehensive view of the interactions between proteins that are involved in various cellular processes. **(C)** A TCM-component-target-disease network: The TCM-component-target-disease network is a graphical representation of the relationships between the active ingredients in *Gardenia*, *Artemisia annua*, and *Honeysuckle*, and the diseases they are believed to treat. The edges in the network represent potential interactions between the different nodes, providing valuable insights into the complex interactions between different components of TCM and their biological targets.

After the selected RDN target genes and SALI target genes were introduced into the Venn chart preparation software Venn 2.1, 96 commonly used target genes were obtained and used as target genes for disease prediction for further research (**Figure 1A**).

##### 2.4.1.4 Protein-protein interaction network construction and key target screening

We used the STRING database (https://string-db.org/cgi/input.pl) to create a PPI network by inputting the common targets of RDN and SALI. The PPI network was constructed with “Homo sapiens” as the biological species and consists of 96 nodes and 853 edges, with an average degree of 17.8 (**Figure 1B** shows the PPI network diagram exported from the STRING website). We imported the TSV files obtained from the STRING database into the Cytoscape 3.9.0 software and performed topological analysis to obtain the core genes (**Table 1**; **Figure 1B**).

**Table 1:** Shared key targets of SALI and RDN.

Target	Degree value
TNF	65
AKT1	65
IL6	58
MAPK3	56
CXCL8	46

On this basis, we integrated the shared targets of RDN and SALI into a library (https://string-db.org/cgi/input.pl) to establish the PPI network. The network comprised of 96 nodes, 853 edges, and 17.8 averages (**Figure 1B**). The documents of TSV were input into Cytoscape 3.9.0, and a new core PPI network was obtained.

##### 2.4.1.5 Construction of TCM–compound–target network

The files of RDN active ingredients and corresponding targets, as well as the attribute files, are imported into the cytoscape software to obtain the drug-component-target network diagram, and acquire the key active ingredients of RDN according to the descending order of the number of targets corresponding to the active ingredient (Degree value).

This study aimed to consider TCM, active components, and shared targets as the research objects and used Cytoscape 3.9.0 to carry on the TCM–compound–target network diagram (**Figure 1C**). The main components selected from the five components from high to low are listed in **Table 2**.

**Table 2:** Key compounds of SALI and RDN.

Compound	Degree value
quercetin	504
kaempferol	402
beta-sitosterol	279
Stigmasterol	227
Mandenol	202

##### 2.4.1.6 Gene oncology and Kyoto encyclopedia of genes and genomes enrichment analysis

We used the David bioinformatics resource version 6.8 (https://david.ncifcrf.gov/home.jsp) to perform enrichment and analysis of GO and KEGG. GO was analyzed in terms of biological processes (BP), cellular components (CC), and molecular functions (MF), with a p-value cut-off of 0.05 and a q-value cut-off of 0.05, while keeping the other settings at their default values. For KEGG analysis, we selected a modified p-value cutoff of <0.05 and used the ggplot2 software in R language for visualization.

##### 2.4.1.7 Molecular docking analysis

The chemical constituents of the target compounds were obtained by PubChem screening, and the preliminary molecular simulation was carried out on RCSB PDB (www.rcsb.org/). The aim of this study was to achieve protein complexes that possess distinct functions by utilizing techniques such as water molecule elimination, hydrogenation, amino acid alteration, energy optimization, and adjustment of electric field parameters.

For interaction between Bax (4ZIE) and mTOR (4DRI), pymol.exe software was used to dehydrate the target proteins and remove the original ligands. ZDOCK online server (http://zdock.umassmed.edu/) was used for protein-protein docking. The pymol.exe software was used to analyze the interaction of its binding mode.

##### 2.4.1.8 Molecular dynamics simulation

Based on the docking results, the quantization software Orca was used to optimize the quantum chemistry of small molecules beta-sitosterol, corymbosin, beta_sitosterol and Stigmasterol under the condition of B3LYP/6-31G*. It involves the correction of bond length, bond Angle, dihedral Angle, and the calculation of fixed charge. The starting structure was created by choosing the best docking results and using amber14sb for the protein and Gaff2 for the small molecule. The transferable intermolecular potential 3P (TIP3P) water model was used to add solvent and sodium ions were added for balance. During the simulation, PME was used to calculate electrostatic interactions and energy minimization was performed using the steepest descent method for up to 50,000 steps. The Coulomb and van der Waals cut-off distances were both set to 1.4 nm. The system was then equilibrated using NVT and NPT before running a 50 ns molecular dynamics simulation at room temperature and pressure.

#### 2.4.2 Molecular biological experiments

##### 2.4.2.1 Immunofluorescence

After the cells nearly reached confluence on pretreated cover glasses, the glasses were removed and the cells were washed twice with phosphate-buffered saline (PBS). The cells were then fixed in 4% paraformaldehyde for 15 min, followed by permeabilization using 0.3% Triton X 100 for 30 min. To block the cells, 5% bovine serum albumin was used for 30 min at 37°C, and the primary antibody was added overnight at 4°C. The next day, the cells were washed with PBS and incubated with a secondary antibody conjugated to a fluorochrome for 60 min at room temperature. Images were captured using an Olympus camera and software, and the experiment was blinded to both the operator and analyzer. Quantification was performed using ImageJ software V1.8.0.112 (Bethesda, USA). Anti-cleaved-caspase-3 antibody (1:500), anti-anti-mTOR antibody (1:500), and anti-Bax antibody (1:1000) were used in this method.

##### 2.4.2.2 Western blotting

WB was performed as described previously (17). The primary antibodies used were anti-p-AKT1 antibody (1:500), anti-AKT1-antibody (1:1000), anti-anti-mTOR antibody (1:500), anti-Bax antibody (1:1000), anti-cleaved-caspase-3 antibody (1:500), anti-caspase-3 antibody (1:1000), anti-cleaved-caspase-9 antibody (1:500), and anti-caspase-9 antibody (1:1000). Proteins levels were analyzed by ImageJ software. The target protein levels were normalized to GAPDH, and the radioactivity was compared to that of the control.

##### 2.4.2.3 Co-immunoprecipitation

A total of 200 µg of HUVECs lysate was treated with either control mouse IgG or Bax-specific antibody for 4 h at low temperature. Furthermore, 50 µL of pre-equilibrated protein A/G agarose beads (50% slurry) were introduced to the mixture, and the solution was placed in a shaker incubator overnight at 4°C. After the incubation, the beads were centrifuged and washed three times with lysis buffer. The elution was carried out using 1X Laemmli buffer, and the Bax protein was analyzed by WB analysis from the elution content. The protocols for SDS-PAGE and WB have been previously described (18).

##### 2.4.2.4 ELISA

An ELISA kit was used to determine the levels of TNF-α in the serum of mice, following the manufacturer’s protocols. The plates were then read using a microplate reader at OD 450 nm and measured with an Epoch microplate spectrophotometer (BioTek, VT, USA). Each sample was tested in triplicate to ensure accuracy.

##### 2.4.2.5 Wet-to-dry weight ratio of the lung

At the designated time for collecting tissue samples, the wet weight of the right lung tissue from mice was measured, followed by drying the right lung in an incubator set at 65°C. After 24 h, the right lung was extracted and weighed to determine its dry weight, which was then used to calculate the wet-to-dry weight ratio of the lung.

##### 2.4.2.6 Hematoxylin-eosin staining

The tissue was preserved using paraformaldehyde, then embedded in paraffin and sliced into 5-μm–thick sections before being stained with HE. The severity of the lung tissue’s pathological injury was evaluated based on the presence of pulmonary interstitial edema and alveolar hemorrhage. The injury scores ranged from 0 (normal) to 4 (extremely severe), and the total score was calculated.

##### 2.4.2.7 Immunohistochemistry

IHC was performed according to the manufacturers’ protocols. The glass slide was dyed on the back, dehydrated to remove impurities, and cured with antigen repair solution. Each was equipped with the corresponding positive and negative controls. The results showed that F4/80 was obviously expressed in the lungs of rats. The p-AKT1 monoclonal antibody (1:500) and F4/80-monoclonal antibody (1:1000) were used in this method.

##### 2.4.2.8 Flow cytometry analysis

Macrophage polarization was detected by flow cytometry. RAW264.7 cells were washed twice in PBS. Then, 3 μ l of PE-anti-mouse CD86 antibody and 3 μl of FITC-anti-mouse CD206 antibody were treated twice in 100 μl of once-binding buffer, then 3 μl of PE-anti-mouse CD86 antibody and 3 μl of FITC-anti-mouse CD206 antibody were treated at room temperature in the dark for 15 min. After incubation, 400 μl of combined buffer was added, and the cells were analyzed by flow cytometry (BD, Franklin Lakes, NJ, USA). Q1+Q2 quadrant represents CD86 positive cells, and Q2+Q3 represents CD206 positive cells.

Apoptosis was detected by flow cytometry. The RAW264.7 cells were washed twice with PBS and resuspended in 100 mL 1x binding buffer. Then, 2.5 μl Annexin-V-FITC and 2.5 μl PE were cultured in the dark box at room temperature for 15 min. In addition, 400 μl of additional adhesive buffers were added to the mixture and analyzed by flow cytometry (BD, Franklin Lakes, NJ, USA). The apoptosis of each group at different time points was detected by PE and Annexin-V assay.

##### 2.4.2.9 JC-1

JC-1 appears as a red fluorescent aggregate in healthy mitochondria. However, with the decrease of membrane potential, JC-1 became a monomer and shows green fluorescence. The digested cells were seeded into six-well plates. After 24 h of stimulation with RDN and LPS, the culture medium was removed, and the positive control group carbonyl cyanide 3-chlorophenylhydrazone (CCCP) staining solution (diluted 1:1000 cell medium) was added first. After incubation at 37°C for 20 min, JC-1 staining solution was added (prepared according to the manufacturer’s instructions). The mixture was thoroughly mixed and incubated at 37°C for 20 min. During incubation, the JC-1 staining buffer was prepared in a 1:4 dilution according to the manufacturer’s instructions. At the end of the incubation period, the supernatant was removed and washed twice with JC-1 staining buffer. The cell culture medium was added, and the final results were observed under a laser confocal microscope.

##### 2.4.2.10 Electron microscopy

The freshly collected lung tissues were trimmed to a size of 1 mm^3^ and fixed in an electron microscopy fixative solution at room temperature in the dark for 2 h. The cell digestion reaction was terminated by adding complete medium, and the cell suspension was centrifuged at 100g for 2 min. The supernatant was discarded, and the electron microscopy fixative was added to make the cells completely suspended in the fixative. Then, the cells were dehydrated, displaced, embedded, and sectioned in a gradient of 50 nm). This was followed by staining with saturated uranyl acetate and lead staining solution, and the section was finally observed under a transmission electron microscope.

### 2.5 Data processing and statistical analysis

Data processing and statistical analysis were conducted using SPSS 22.0 (IBM Corporation, Armonk, NY, USA) with data obtained from three independent experiments. T-test was used to compare two groups, while ANOVA was used to compare multiple groups. Results were showed as mean ± standard deviation (SD). Furthermore, post-hoc tests were used to assess the statistical significance among means. Prism 8.0 (GraphPad Software, USA) was used to visualize.

## 3 Results

### 3.1 GO and KEGG results for the shared targets of RDN and SALI

A total of 1634 GO entries were found to be enriched, with 217 related to cellular components (CC). These CCs were primarily located in various regions such as the plasma membrane, extracellular space, cytoplasm, complex macromolecules, perinuclear region of the cytoplasm, extracellular exosomes, caveolae, and ficolin-1-rich granule lumen. Additionally, 58 biological processes (BP) were identified, which played a role in regulation and participated in various activities such as positive regulation of smooth muscle cell proliferation, response to lipopolysaccharides, inflammatory response, positive regulation of gene expression, positive regulation of interleukin-8 production, response to hypoxia, positive regulation of ERK1 and ERK2 cascade, protein phosphorylation, and LPS-mediated signaling pathways.

**Figure 2:**
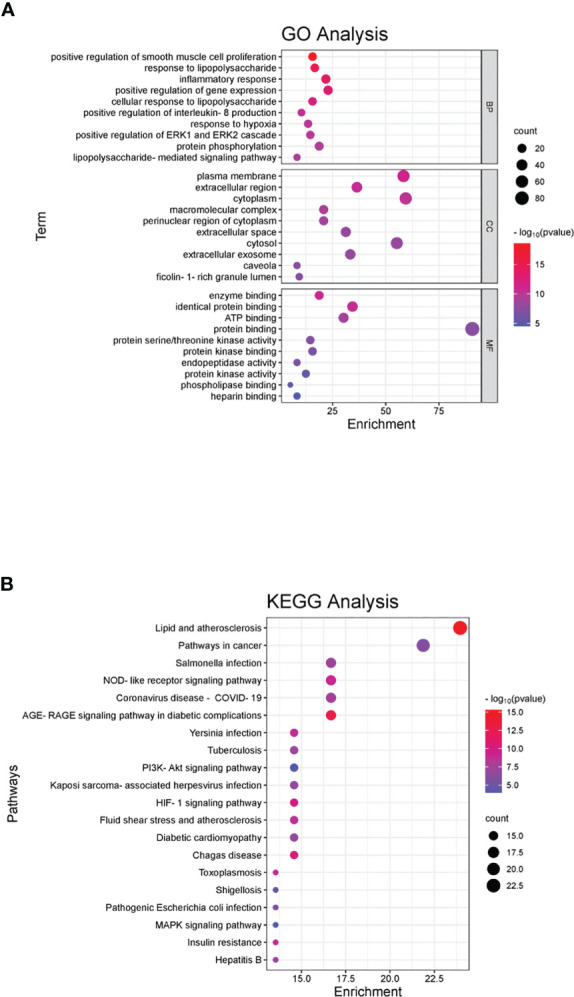
GO and KEGG pathway enrichment of the shared genes. **(A)** In the GO analyses, we observed significant enrichment in certain categories, with the x-axis representing the categories and the y-axis displaying the target genes. The color of each category indicates its p-value, with red indicating a smaller p-value and blue indicating a larger p-value. **(B)** In the KEGG pathway enrichment analyses, we identified several main pathways, with the y-axis displaying the pathway names and the x-axis showing the counts of target symbols in each pathway. The color of each pathway represents its p-value, with red indicating a smaller p-value and green indicating a larger p-value.

There were 94 molecular functions (MF), mainly including enzyme binding, identical protein binding, ATP binding, protein binding, protein serine/threonine kinase activity, protein kinase binding, peptide chain endonuclease activity, protein kinase activity, phospholipase binding, and heparin binding. We selected the top 10 enrichment items based on their P values and used the ggplot2 package in R language to create a GO analysis bubble plot, taking into account the P value, Q value, and number of enriched genes in each item. The plot displays the number of targets on the x-axis, with BP, CC, and MF on the left; the P value is represented by color. Red indicates a smaller P value, while blue indicates a larger P value (**Figure 2A**).

KEGG was mainly enriched in the pathways of lipids and atherosclerosis, cancer, AGE-RAGE signaling pathway in diabetic complications, NOD-like receptor signaling pathway, coronavirus disease (COVID-19), *Salmonella* infection, Chagas disease, HIF-1 signaling pathway, plague infection, fluid shear stress, atherosclerosis, tuberculosis, Kaposi’s sarcoma-associated herpesvirus infection, diabetic cardiomyopathy, PI3K-Akt signaling pathway, insulin resistance, toxoplasmosis, hepatitis B, pathogenic *E. coli* infection, shigellosis, and MAPK signaling pathway. The horizontal axis represents the number of enriched genes, the left side represents the pathway name, and the color represents the p-value. More red than blue represents a higher significance (**Figure 2B**).

### 3.2 Molecular docking analysis between the key compounds of RDN and the key shared targets

**Figure 3:**
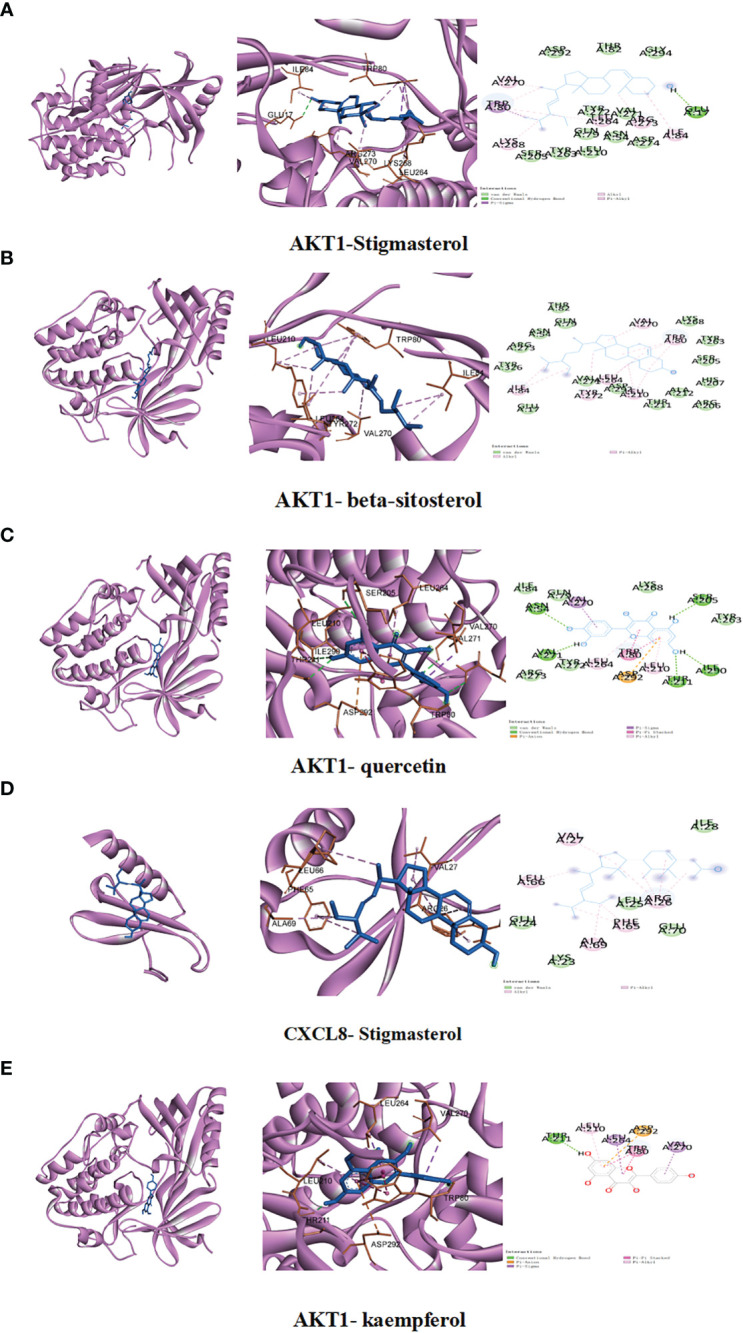
We conducted studies to investigate the interactions between specific ingredients and targets. **(A)** AKT1 with stigmasterol; **(B)** AKT1 with beta-sitosterol; **(C)** AKT1 with quercetin; **(D)** CXCL8 with stigmasterol; **(E)** AKT1 with kaempferol.

Molecular docking was conducted to confirm the binding activity of stigmasterol, kaempferol, quercetin, beta-sitosterol, and corymbosin with key targets SRC, TNF, CASP3, GAPDH, and ERBB2. The outcome showed that all the compounds exhibited favorable binding activity. The top five binding energy results of each target protein were selected, plotted, and analyzed (**Table 3**; **Figure 3**). Among the top five binding abilities, there were four groups of different active components with the same shared target—AKT1. These results suggest that AKT1 likely plays a key role in RDN treatment of SALI.

**Table 3:** Molecular docking binding energy.

ID	Target	Compound	Binding ability(kcal/mol)
1	AKT1	Stigmasterol	-11.3
2	AKT1	beta-sitosterol	-11.1
3	AKT1	quercetin	-9.8
4	CXCL8	Stigmasterol	-9.6
5	AKT1	kaempferol	-9.5
6	TNF	Stigmasterol	-9.4
7	CXCL8	beta-sitosterol	-9.2
8	AKT1	Corymbosin	-9.1
9	TNF	quercetin	-9.1
10	MAPK3	quercetin	-9
11	TNF	kaempferol	-8.8
12	MAPK3	kaempferol	-8.6
13	TNF	Corymbosin	-8.5
14	MAPK3	Stigmasterol	-8
15	CXCL8	kaempferol	-7.4
16	MAPK3	beta-sitosterol	-7.3
17	CXCL8	Corymbosin	-7.1
18	CXCL8	quercetin	-7.1
19	IL6	kaempferol	-7
20	IL6	quercetin	-6.8
21	MAPK3	Corymbosin	-6.8
22	IL6	Corymbosin	-6.7
23	IL6	beta-sitosterol	-6.7
24	IL6	Stigmasterol	-6.5
25	TNF	beta-sitosterol	-6.2

### 3.3 Molecular dynamics simulation presented the possible link between RDN and AKT1

**Figure 4:**
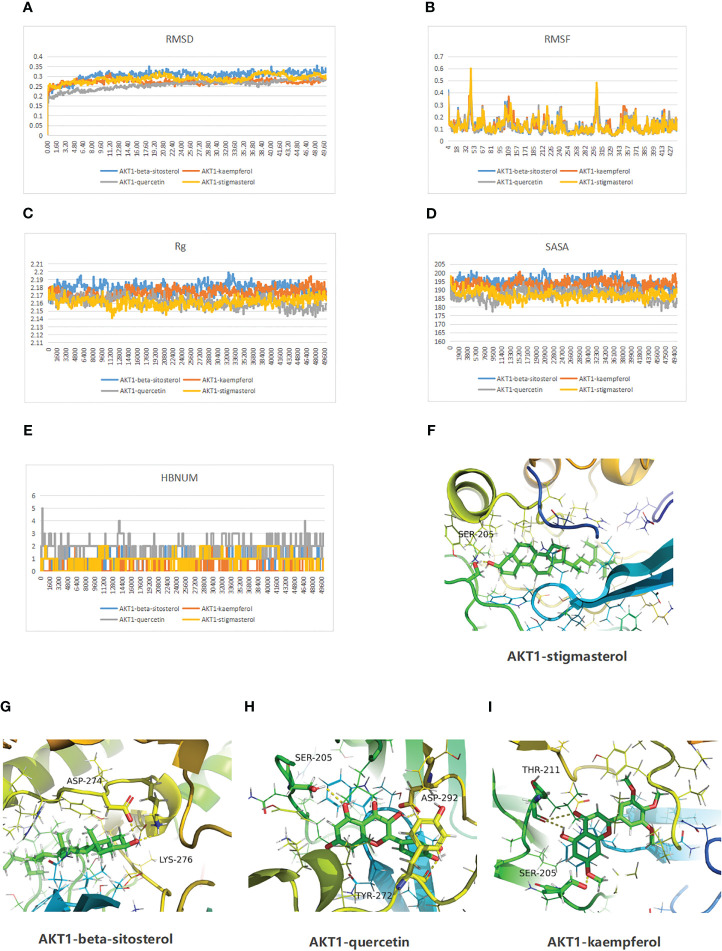
Molecular dynamics simulation results of the complexes of the active ingredients of RDN and AKT1 proteins are as follows. **(A)** RMSD; **(B)** Protein RMSF; **(C)** Rg; **(D)** SASA; and **(E)** HBNUM. Besides, the molecular docking steady state was also displayed: **(F)** AKT1-stigmasterol; **(G)** AKT1-beta-sitosterol; **(H)** AKT1 with quercetin; and **(I)** AKT1 with kaempferol.

We delved deeper into the stability and kinetics of the complexes when they were in a water-based solution. The root mean square deviation (RMSD) is computed once the desired shape and original shape have been minimized. As shown in **Figure 4A**, after the initial transient perturbation of protein and ligand, complexes 1–4 can basically proceed to steady state at 10 ns, with a value approaching 0.25 nm and a fluctuation range of 0.05 nm. The root mean square fluctuation (RMSF) can indicate the variability observed during the simulation. The consistency of protein movements across all simulations enhances the credibility of the model. All four complexes fluctuated more than 0.25 nm at 4,41, 67, 220, 243, 300, 301, and 307 ns (**Figure 4B**). Rg measures the density of a protein, which will remain relatively stable if the protein fold is stable. If a protein is unfolded, its Rg value will change over time. As shown in **Figure 4C**, all four groups of proteins folded stably. **Figure 4D** illustrates the use of solvent accessible surface area (SASA) to examine the protein surface’s solvent accessibility, revealing a consistent decline in the protein’s SASA between 0 and 50 ns, indicating a gradual contraction of the protein. Additionally, the simulation calculated the hydrogen bond number (HBNUM), which represents the number of hydrogen bonds between protein ligands. The AKT1-beta-sitosterol, AKT1-corymbosin, and AKT1-stigmasterol group remained stable at 0–2 contacts, while the AKT1-quercetin group showed fluctuations between 1 and 3 contacts (**Figure 4E**). The molecular docking steady state of the complexes was presented also (**Figure 4F–I**).

### 3.4 RDN could improve survival rate in SALI mice model

**Figure 5:**
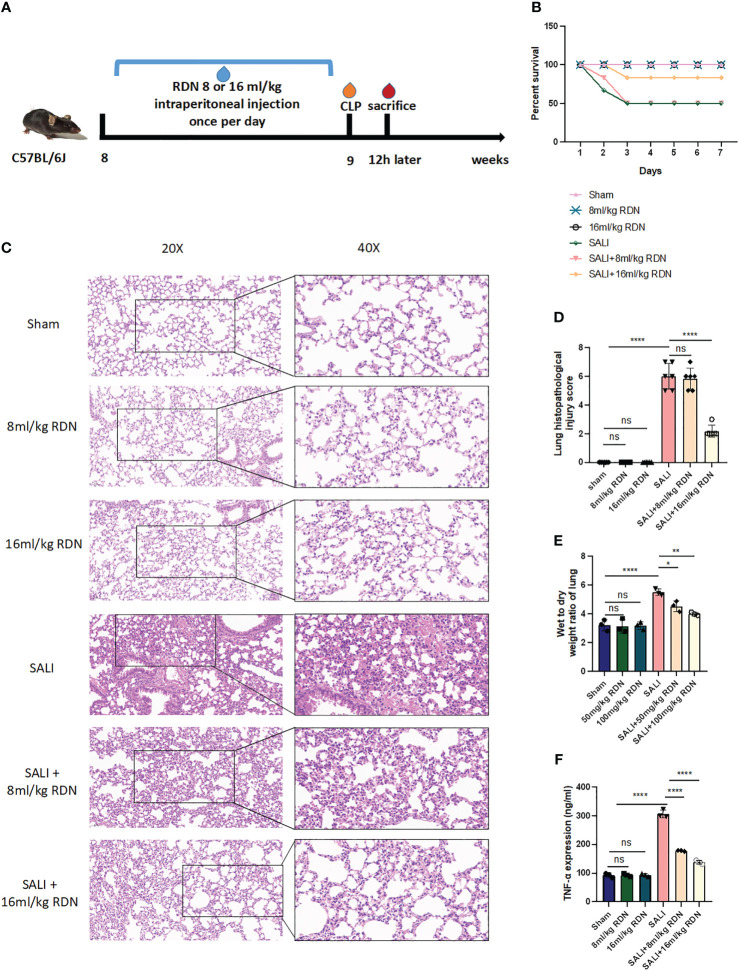
RDN could attenuate the lung injury in the sepsis mice model. **(A)** A visual representation of animal testing experiments. **(B)** The percentage of survival for various groups including a control group, two groups given different doses of RDN, a SALI group, and two groups given RDN after inducing CLP. **(C)** Histopathological examination was conducted to assess the lung’s condition. **(D)** A score was assigned to evaluate the severity of lung damage. **(E)** The ratio of wet-to-dry lung tissue was measured. **(F)** The level of TNF-α was determined using ELISA. The data represents the mean ± standard deviation of results from three separate experiments, with three samples per group. Images were magnified at 20× and 40×, with a scale bar of 50 μm and 20 μm, respectively. *P<0.05, **P<0.01, and ****P<0.001; ns means there is no statically significant difference.

To further confirm the efficacy of RDN *in vivo*, the study divided mice into six groups: sham group, RDN (8 mL/kg or 16 mL/kg) group, SALI group, and SALI+RDN (8 mL/kg or 16 mL/kg) group. The RDN group received the indicated concentration *via* intraperitoneal injection once daily for 7 days. The SALI+RDN group received RDN *via* intraperitoneal injection 12 h after the 7-day injection (**Figure 5A**). The research tracked the number of mice that survived for 7 days after undergoing a CLP operation. As shown in **Figure 5B**, while the sham and RDN groups survived until the end of the observation period, half of the mice in the SALI group died within 3 days. The survival rate of the mice in the SALI group was lower than that of the sham group (P<0.05). The mice in the SALI+16 mL/kg RDN group had a significantly better survival rate than the SALI group (P<0.05). However, there was no significant difference in survival rate between the CLP+8 mL/kg RDN group and the SALI group (P>0.05).

### 3.5 RDN could alleviate lung injury *in vivo*

Six hours after the operation, mice in the sham, 8 mL/kg RDN, and 16 mL/kg RDN groups did not show any signs of lung congestion, bleeding, or inflammation. However, mice in the SALI group had severe lung damage, including hyperemia, hemorrhage, edema, and inflammation (**Figure 5C**). Mice in the SALI+8 mL/kg RDN group had less severe lung damage than the SALI group, while the SALI+8 mL/kg RDN group did not show any significant improvement. The lung histopathological injury scores of mice in the Sham group, 8 mL/kg RDN group, 16 mL/kg RDN group, SALI group, SALI+8 mL/kg RDN group, and SALI+16 mL/kg RDN group were 0.333 ± 0.21, 0.167 ± 0.167, 0.2 ± 0.2, 6.0 ± 0.365, 5.833 ± 0.307, and 2.167 ± 0.167, respectively (**Figure 5D**). The lung injury scores were significantly different between the groups, with the SALI group having the highest score and the sham, 8 mL/kg RDN, and 16 mL/kg RDN groups having similar scores. The SALI+16 mL/kg RDN group had a significantly lower score than the SALI group, while the SALI+8 mL/kg RDN group did not show any significant improvement. Hence, we considered 8 mL/kg/day RDN as the effective concentration and put it into following experiments. Besides, RDN can indeed ameliorate the functional damage of other organs (myocardium, liver, and kidney) to some extent (**Supplementary Data**).

### 3.6 RDN could attenuate pro-inflammatory factor TNF-α and wet-to-dry weight ratio of the lung *in vivo*

**Figure 5E** shows that the wet-to-dry weight ratio of lung tissue in the model group was significantly higher than that in the sham group (P<0.001). However, the wet-to-dry weight ratio of lung tissue in the SALI+RDN group was significantly lower than that in the model group (P<0.001). In addition, the group that received a higher dosage of RDN demonstrated a notable reduction in the ratio of wet-to-dry weight in lung tissue compared to the group that received a lower dosage of RDN (P<0.01).

To investigate the role of TNF-α in sepsis, we measured the levels of this pro-inflammatory cytokine in the serum of mice using ELISA. As shown in **Figure 5F**, the levels of TNF-α in the SALI group were higher than those in the sham group. However, RDN treatment significantly reduced the level of TNF-α in the SALI+RDN group (P<0.05).

### 3.7 RDN could relieve PMECs apoptosis and vascular leakage *in vivo*

**Figure 6:**
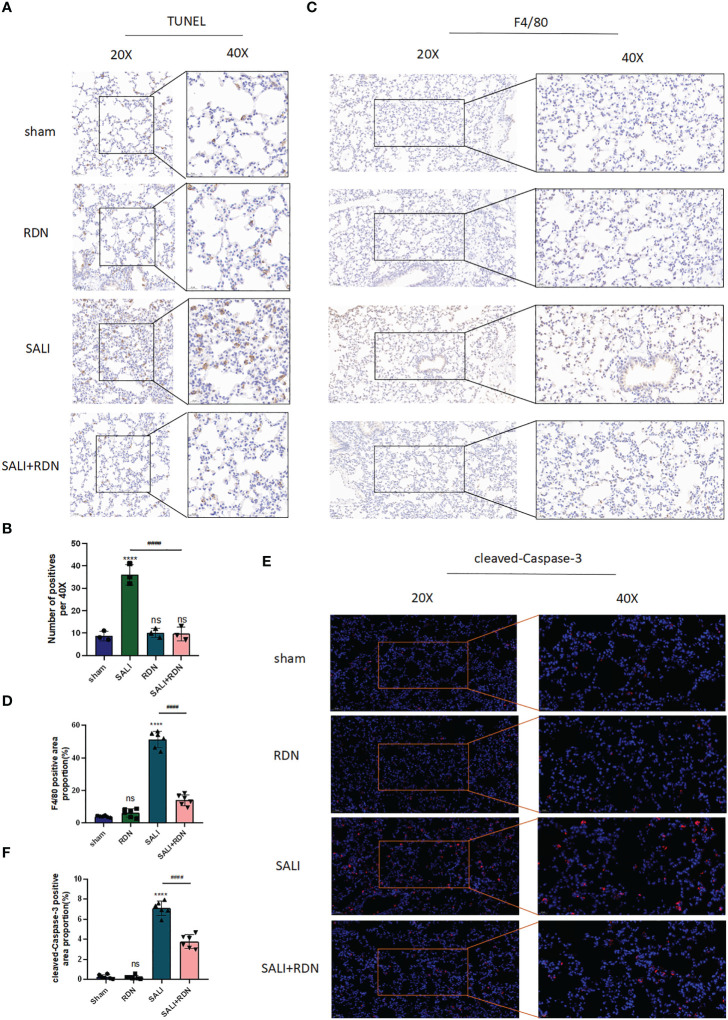
RDN could protect the PMECs barrier function. **(A, B)** TUNEL assay was used to detect the level of apoptosis. **(C, D)** IHC was used to investigate the level of F4/80. **(E, F)**, and IF was used to detect the level of cleaved-caspase-3. ****P<0.001, “*” indicates that there is a significant difference compared to the sham group. ^####^P<0.001, ns means there is no statically significant difference.

To confirm that RDN is effective in treating SALI in mice, we used IHC to examine the amount of the macrophage biomarker protein F4/80 and TUNEL to determine the level of cell apoptosis. Our results showed that the number of positive cells was significantly increased in the SALI group compared to the sham group (P<0.001), but was significantly decreased in the SALI+RDN group compared to the SALI group (P<0.001) (**Figures 6A, B**). Similarly, the protein expression level of F4/80 was significantly enhanced in the SALI group compared to the sham group (P<0.001), but was critically decreased in the SALI+RDN group compared to the SALI group (P<0.001) (**Figures 6C, D**). There was no significant difference between the sham group and RDN group (P>0.05) for both parameters. However, the SALI group showed a significant increase in the protein expression level of cleaved-caspase-3 compared to the sham group (P<0.001). On the other hand, the SALI+RDN group showed a significant decrease in cleaved-caspase-3 expression compared to the SALI group (P<0.001) (**Figures 6E, F**). The sham group and RDN group did not differ statistically (P>0.05).

### 3.8 RDN could regulate AKT1-mTOR-Bax signaling pathway in PMECs *in vivo*

**Figure 7:**
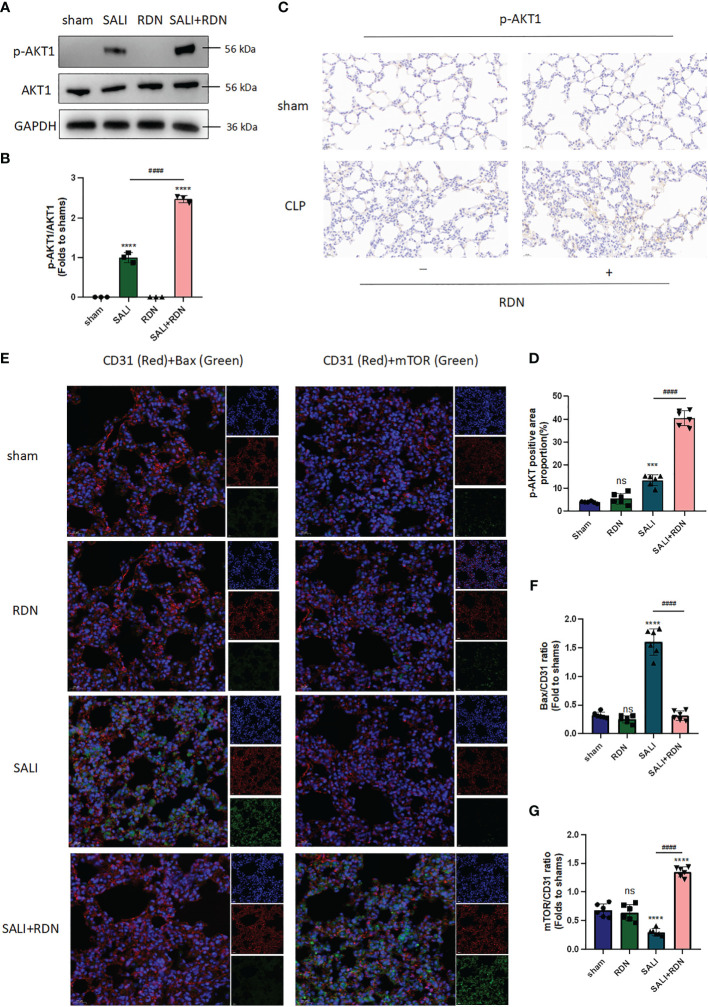
RDN could improve the AKT-mTOR pathway and reduce the Bax expression level. **(A, B)** WB was applied to evaluate the expression ratio of p-AKT/AKT. **(C, D)** IHC was used to measure the expression level of p-AKT. **(E, G)** IF was employed to investigate the level of Bax and mTOR in endothelial cells (CD31^+^). ****P<0.001, ***P<0.005, “*” indicates that there is a significant difference compared to the sham group. ^####^P<0.001, ns means there is no statistically significance.

The results from **Figures 7A, B** suggest that the SALI group had significantly higher levels of p-AKT1/AKT1 expression in the lung tissue than the sham group (P<0.001). Additionally, the SALI+RDN group had even higher levels of p-AKT1 expression in the lung tissue than the SALI group (P<0.001). These findings were consistent with both western blot (WB) and immunohistochemistry (IHC) analyses (**Figures 7C, D**). The SALI group had a slight increase in p-AKT1 expression in lung tissue compared to the sham group, but the SALI+RDN group had significantly higher levels of p-AKT1 expression (P<0.001). Furthermore, the immunofluorescence (IF) analysis in **Figures 7E–G** revealed that the Bax/CD31 ratio increased in the LPS group, while the mTOR/CD31 ratio decreased. However, RDN could reverse these effects by decreasing the Bax/CD31 ratio and increasing the mTOR/CD31 ratio.

### 3.9 RDN could regulate endothelial cell apoptosis through activating the AKT-mTOR signaling pathway *in vitro*

**Figure 8:**
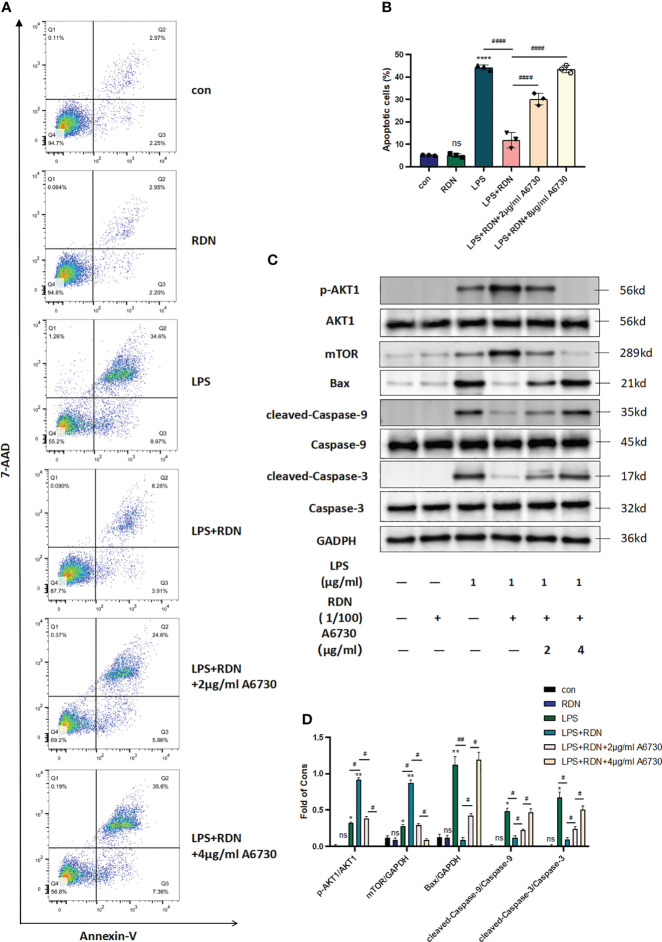
RDN may have a protective effect on endothelial cells by reducing apoptosis. **(A, B)** Flow cytometry analysis revealed a lower proportion of apoptotic cells in the RDN group than the LPS group. **(C, D)** Western blot analysis showed a decrease in the level of apoptosis-related proteins in the RDN group. These results support the potential therapeutic use of RDN in preventing endothelial cell apoptosis. The data presented are the mean ± SD of three independent experiments with three replicates per group, and statistical analysis indicated a significant difference between the RDN group and the LPS group. ****P<0.001, ***P<0.005, **P<0.01, *P<0.05, “*” indicates that there is a significant difference compared to the sham group. ^####^P<0.001, ^##^P<0.01, ^#^P<0.05. ns means there is no statically significant difference.

The level of apoptosis was measured using flow cytometry, and the apoptosis rate was determined by analyzing the Q2+Q3 regions of the 7-AAD and Annexin-V assay. The results in **Figures 8A, B** indicate that LPS significantly increased the level of apoptosis (P<0.001), while RDN was found to be an effective means of alleviating it (P<0.001). The proportions of apoptotic cells were 5.083 ± 0.115, 5.063 ± 0.494, 44.31 ± 0.684, 11.82 ± 1.998, 30.19 ± 1.513, 4.44 ± 0.711, and 43.56 ± 0.956 in the control group, RDN group, LPS group, LPS+RDN group, LPS+RDN+2 μg/mL A6730 group, and LPS+RDN+4 μg/mL A6730 group, respectively.

**Figures 8C, D** indicated that the levels of p-AKT1/AKT1 and mTOR/GAPDH increased slightly following stimulation with LPS (P<0.001). However, the LPS+RDN group exhibited significantly higher levels of p-AKT1/AKT1 and mTOR than the LPS group (P<0.001). Additionally, the expression of apoptosis-related proteins cleaved-caspase-3/caspase-3, cleaved-caspase-9/caspase-9, and Bax in the LPS group was higher than that in the control group, while the LPS+RDN group showed a significant reduction in the expression of these proteins compared to the LPS group (P<0.05). No significant difference was observed between the RDN group and the control group (P>0.05). After treatment of the LPS group with AKT specific inhibitor A6730, the level of mTOR decreased with the decrease of p-AKT1/AKT1 ratio, and the levels of apoptosis-related proteins cleaved-caspase-3/caspase-3, cleaved-caspase-9/caspase-9, and Bax increased.

### 3.10 RDN could alleviate endothelial cells’ mitochondrial damage *in vitro*

**Figure 9:**
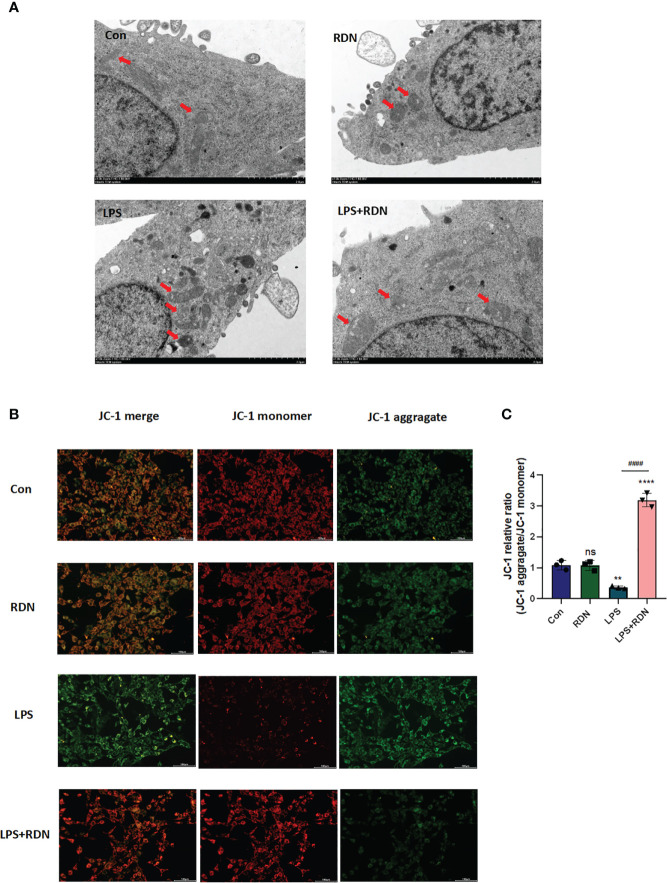
RDN could attenuate mitochondrial damage caused by LPS *in vitro.* **(A)** Transmission electron microscopy was used to visualize changes in mitochondrial structure **(B, C)** JC-1 was used to detect changes in mitochondrial membrane potential. The data shown are the average value plus or minus the standard deviation, with three samples in each group. The results are representative of three separate experiments. The statistical significance is indicated as **P<0.01, ****P<0.001, “*” indicates that there is a significant difference compared to the sham group. ^####^P<0.001. ns means there is no statically significant difference. The images were taken at a magnification of 7000× and the scale bar represents a length of 2 µm.

Under the electron microscope, the mitochondrial membrane and cristae structures in both the control and RDN groups were clearly defined and undamaged, with no evidence of split mitochondria. Conversely, in the LPS group, a significant number of mitochondria were observed to be undergoing division or had recently completed division, with incomplete mitochondrial membrane structures and absent cristae, suggesting an increase in abnormal division. However, in the LPS+RDN group, the majority of mitochondrial structures remained intact, with only a few instances of broken or absent cristae (**Figure 9A**).

Maintaining a normal mitochondrial membrane potential (ΔΨm) is essential for proper mitochondrial function and cellular health. A stable ΔΨm contributes to normal physiological function of cells. However, during injury or apoptosis, a decrease in mitochondrial membrane potential can occur. To investigate the impact of RDN on mitochondria, cells induced by LPS were assessed using JC-1, a cationic dye that aggregates in mitochondria. At low concentrations, JC-1 exists as a monomer and emits green light, while at high concentrations, it forms multimers and emits red light. Laser confocal observation revealed that JC-1 staining showed a significant increase in the ratio of green to red light, indicating a weakened ΔΨm in the LPS group compared to the control group (**Figures 9B, C**). However, co-treatment with LPS+RDN reversed these results, suggesting that RDN improved the LPS-induced decrease in ΔΨm level. Notably, there was no significant difference in the ΔΨm between the RDN group and control group.

### 3.11 mTOR could directly bind to Bax *in vitro*

**Figure 10:**
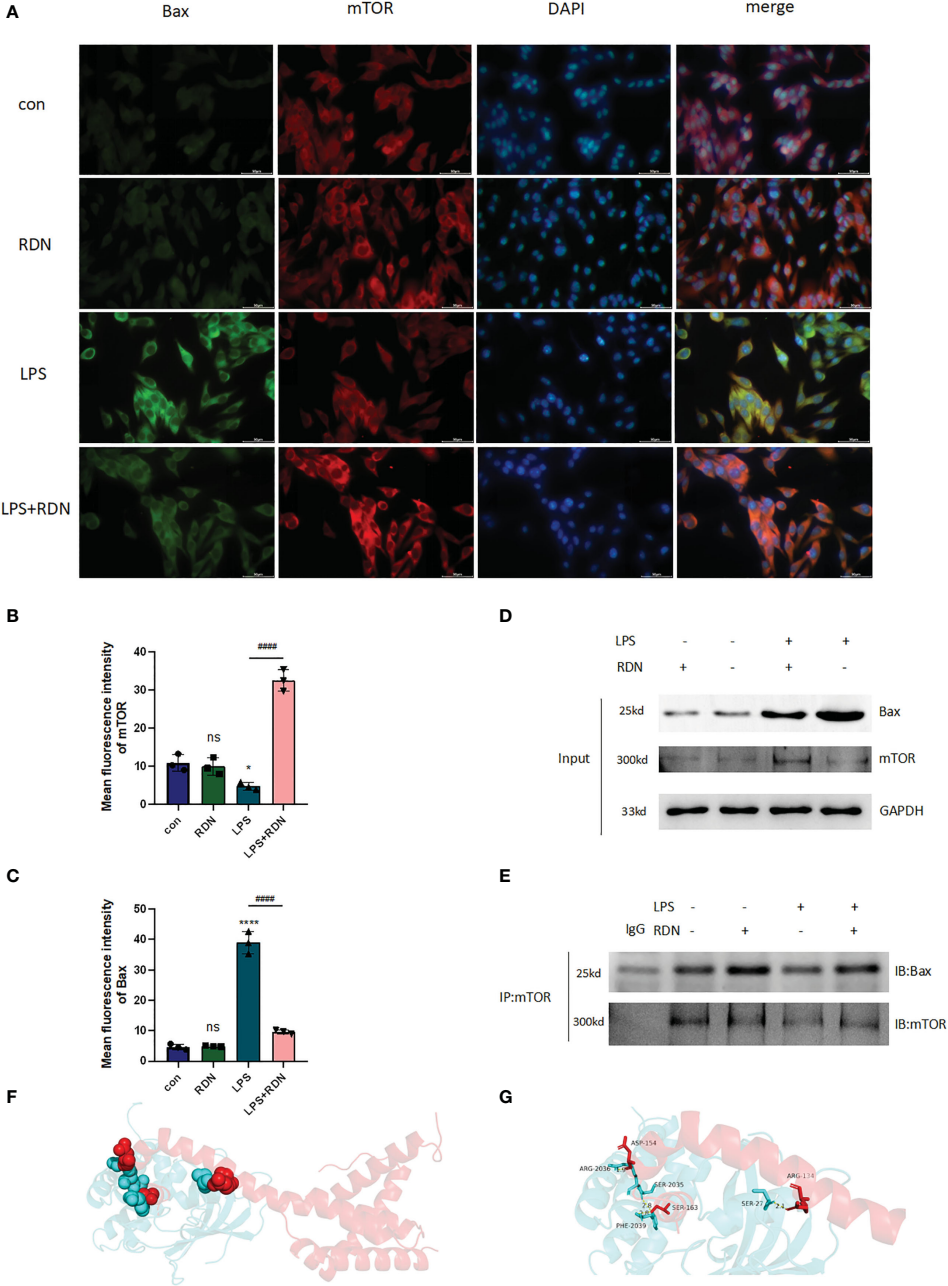
The interaction of Bax and mTOR in LPS-induced HUVECs after RDN intervention. **(A–C)** IF was applied to detect the level of Bax and mTOR expression *in vitro*. ^####^P<0.001, ****P<0.001, *P<0.05, “*” indicates that there is a significant difference compared to the sham group. ns means there is no statically significant difference. **(D, E)** CoIP was employed to investigate the interaction of Bax and mTOR and the impact of RDN on the process. The possible interaction between mTOR and Bax was predicted by molecular docking. Based on the docking mode of protein Bax (4ZIE) and protein mTOR (4DRI), where red diagram represents protein Bax and blue diagram represents protein mTOR. **(F)** The locations of docking pockets of the two proteins and their interaction sites and interaction modes. **(G)** The specific amino acid residues at the interaction sites and the bond lengths of hydrogen bonds.

The results of the IF analysis revealed that in the LPS group, the level of Bax was elevated, while the level of mTOR was reduced. However, RDN treatment could reverse these effects, as evidenced by the decreased level of Bax and the increased level of mTOR in HUVECs (**Figures 10A–C**).

To determine whether mTOR interacts with Bax in HUVECs, we conducted CoIP experiments. We first detected the expressions of mTOR and Bax proteins using Input WB (**Figure 10D**). Our results showed that the empty GFP plasmid did not bind to Bax, while GFP-tagged mTOR was observed to bind to Bax in HUVECs. Moreover, interestingly, after RDN intervention, the same quantity of mTOR was able to bind to a greater amount of Bax (**Figure 10E**). Therefore, our study suggests that mTOR likely interacts with Bax in HUVECs and that RDN intervention may enhance this interaction.

Furthermore, molecular docking technology was employed to explore the docking mode of mTOR and Bax. According to the docking results, the two proteins were mainly connected through three docking pockets, all of which were linked by hydrogen bonds (**Figure 10F**). The specific amino acid residues involved in docking and the bond lengths of hydrogen bonds are presented in **Figure 10G**.

## 4 Discussion

In a state of good health, the body triggers immune responses to combat disease-causing agents such as viruses, bacteria, fungi, and parasites. This immune response is crucial to limit the spread of infection and prevent it from becoming more severe (20). If the innate and adaptive immune responses to infection are not properly regulated, the infection can spread and cause damage to multiple organs, ultimately leading to sepsis. The lungs are particularly vulnerable to damage in case of sepsis, and the resulting hypoxia can exacerbate dysfunction in other organs such as the heart, liver, and kidneys. Additionally, dysfunction of pulmonary microvascular endothelial cells during sepsis can contribute to acute pulmonary edema and the development of acute respiratory distress syndrome (ARDS) (2). While current clinical treatments prioritize lung protective ventilation, there is still no definitive drug therapy available. Given that many ARDS survivors experience long-term complications, early recognition of ARDS is crucial, and ongoing research into the underlying mechanisms of lung injury is necessary to develop targeted treatments (21). In this study, the sepsis model was established by CLP, the TNF-α was detected by ELISA to reflect the infection of the animal model (22, 23). HE staining, lung injury index, and lung wet/dry weight ratio showed that the sepsis mouse model in this study successfully induced lung injury (24, 25). In addition, the sepsis model used in this study successfully induced multiple organ dysfunction (myocardium, liver, and kidney). Consequently, our study on the protective effect of RDN was based on the SALI mouse model.

With the development of the concept of suspected sepsis, early identification of sepsis or intervention for infection progression to sepsis can effectively reduce the morbidity and mortality of sepsis (26, 27). The idea of early prevention and blocking of sepsis coincides with the theory of “preventive treatment for disease” of traditional Chinese medicine. This is the original intention of our study to explore the protective effect of RDN on SALI. Thus, we use RDN intervention before CLP, hoping to obtain a drug that can block further organ damage in the period of suspected sepsis, and reduce the incidence of sepsis and infection mortality.

Network pharmacology provides a fresh perspective for the modernization research of TCM, offering a more suitable approach to explore the multi-target effects of this ancient healing method. By analyzing the interrelationships between different components of TCM, studying the biological pathway signals of diseases, and establishing the overall relationship between active ingredients, targets, and diseases, network pharmacology aligns with the holistic view of TCM treatment (28, 29). In our prior research, we investigated the effects of RDN on LPS-induced HUVECs through a combination of network pharmacology and *in vitro* studies, which revealed that RDN has the potential to significantly mitigate LPS-induced apoptosis in HUVECs by activating the AKT pathway (17). In this study, the MDs results of key disease-drug shared targets and key active components in our experiment showed that four of the top five active components of docking binding energy all docked with AKT1, which partly predicted that multiple key active components of RDN could improve SAKI through AKT1. The GO and KEGG results of shared targets showed that AKT1-related PI3K-AKT pathway and protein phosphorylation played an important role in it, and this pathway was closely related to apoptosis. In addition, the ability of RDN to activate PI3K-AKT pathway to improve LPS-induced apoptosis in HUVECs was also demonstrated in our previous study. Therefore, in this experiment, we focused on the protective effect of RDN on SALI using pulmonary vascular endothelial cell apoptosis as the entry point.

The apoptosis of PMECs plays a key role in development of SALI. The study by Gill et al. demonstrated that the dysfunction of pulmonary microvasculature in septic mice is caused by the death of PMECs, which occurs *via* caspase-dependent apoptosis and iNOS/NADPH-oxidase dependent signaling (30). Therapies that focus on addressing PMECs apoptosis have been proven to be effective in improving SALI. One such example is pravastatin, which has demonstrated the ability to enhance survival rates, alleviate lung pathological changes, and reduce pulmonary microvascular permeability in septic mice. This is achieved by improving alveolar endothelial barrier disruption through modulation of the Cav-1/eNOS pathway (31). Heat shock protein A12A (HSPA12A) could protect LPS-induced endothelial dysfunction from hyperpermeability and death through both ERKs and AKT-mediated signaling (32). Above all, prevention of apoptosis of PMECs is a promising means to alleviate the progression of infection to SALI. This time, the research aimed to explore the beneficial impact of RDN on preventing PMECs apoptosis, as a means of protecting against SALI.

The regulation of PI3K-AKT pathway is vital in alleviating the apoptosis of endothelial cells and epithelial cells in lung injury. Research has shown that the ACE2-mediated SARS-CoV-2 infection can induce apoptosis by inhibiting the PI3K-AKT-mTOR pathway in human bronchial epithelial and microvascular endothelial cells (33). However, adipose-derived mesenchymal stem cells-derived exosomes have been found to inhibit lung hemorrhage and edema, and reduce vascular hyper-permeability by activating the PI3K-AKT pathway and protecting endothelial cells (34). In a previous study, it was demonstrated that mTOR can directly bind to Bax in HUVECs, highlighting the importance of studying drugs that can interfere with the PI3K-AKT-mTOR pathway for the prevention and treatment of SALI (18). Therefore, it is very important to study and explore drugs that can interfere with the PI3K/AKT/mTOR pathway for the prevention and treatment of SALI. This study aims to explore the protective mechanism of RDN in the CLP-induced SALI mice model and LPS-induced HUVECs, including its effect on AKT downstream protein mTOR and apoptosis-related Bax.

In this study, significant improvements have been observed in lung tissue damage, apoptosis levels, pro-inflammatory cell infiltration, and survival rates in SALI mice with RDN intervention. These improvements were accompanied by an activated AKT/mTOR signaling pathway and decreased Bax expression in PMECs. Results from WB and flowcytometry showed that RDN could attenuate apoptosis-related proteins and cell apoptosis rates *in vitro*. Furthermore, transmission electron microscopy and JC-1 assay confirmed RDN’s ability to ameliorate mitochondrial damage caused by LPS. As mitochondria play a crucial role in both cellular respiration and apoptosis regulation, these findings provide valuable insights into RDN’s therapeutic potential (35). The apoptosis signaling pathway involves the interaction between BH3 domain-containing Bcl-2 family members such as Bid, Bad, Bim, and Bax family members like Bax and Bak that reside in the cytosol or bind to the mitochondrial outer membrane. The insertion of Bax family members into the mitochondrial membrane causes changes in mitochondrial membrane permeability, loss of transmembrane potential, and subsequent release of cytochrome C (Cytc) and other proteins. The release of Cytc is a critical step in the mitochondrial apoptosis pathway. Released cytochrome C binds to apoptosis-associated factor 1 (Apaf-1) to recruit caspase-9 precursors in the cytoplasm. Activated caspase-9, in turn, activates caspase-3, leading to the induction of apoptosis (36). Next, to further verify the key role of AKT1 in SALI, AKT specific inhibitor A6730 was used to block the AKT signaling pathway. *In vitro*, it was showed that the blocking effect of RDN on apoptosis could be reversed by A6730. That is to say, the AKT pathway plays an essential role in the protective effect of RDN on SALI.

Furthermore, we investigated RDN’s potential mechanism in alleviating PMECs apoptosis. It was showed that RDN significantly reduced the expression of apoptosis-related protein Bax and increased the expression of downstream AKT pathway protein mTOR in LPS-induced HUVECs. Using molecular docking technology, we found that mTOR and Bax have three docking pockets connected by hydrogen bonds. Further CoIP experiments revealed that RDN not only increased the expression level of mTOR but also enhanced its ability to bind to Bax. This may explain why RDN could down-regulate PMECs apoptosis by activating the AKT/mTOR pathway.

However, the pharmacological mechanism of RDN was not verified in animals with endothelial cell-specific AKT1 knockout, but the scientific hypothesis that RDN improves SALI through AKT1-mTOR-Bax pathway was proven to some extent by administering HUVECs the AKT-specific inhibitor A6730 and using CoIP and other research methods, which will not cause too much bias. We will continue to explore the protective mechanism of RDN on sepsis patients in clinical practice, hoping to provide basic support for the application of traditional Chinese medicine in sepsis. Also, drug absorption distribution should be evaluated in the future.

## 5 Conclusions

**Figure 11:**
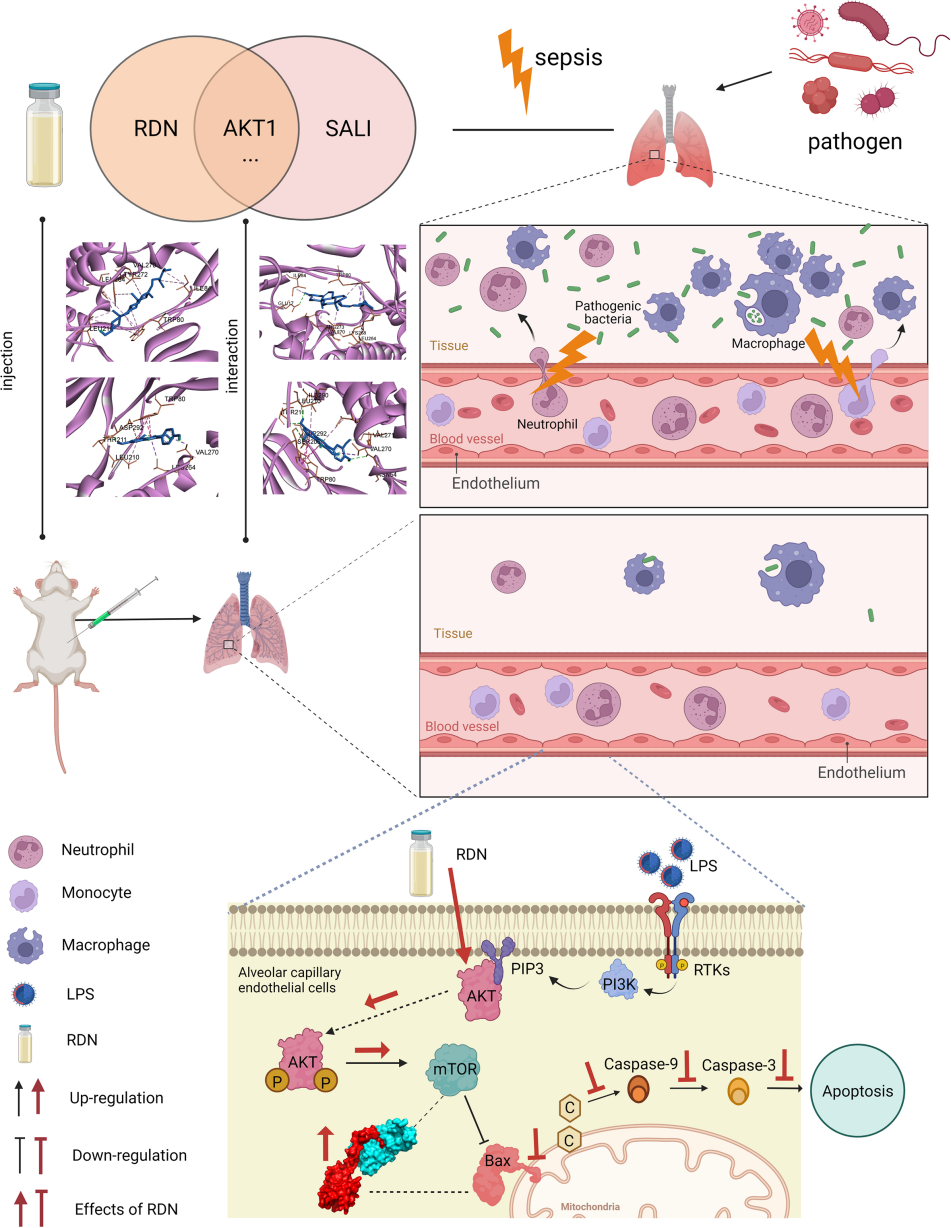
Apoptosis level was essentially enhanced in SALI mice. RDN has obvious therapeutic effect on SALI mice. RDN significantly activated the AKT1/mTOR signaling pathway, decreased Bax level, down-regulated the level of inflammatory factor TNF-α, and significantly decreased PMECs apoptosis. Combining CoIP, IF, and molecular docking studies, we showed that RDN could promote the AKT1/mTOR signaling pathway and attenuate SALI by enhancing the binding capability of Bax and mTOR.

In this study, network pharmacology and experimental models were used to predict and validate the therapeutic effect of RDN. These findings strongly support the protective effect of RDN on SALI by reducing endothelial apoptosis and preserving the PMECs barrier function to prevent immune cell leakage (**Figure 11**).

## Data availability statement

The original contributions presented in the study are included in the article/**Supplementary Material**. Further inquiries can be directed to the corresponding author.

## Ethics statement

The animal study was reviewed and approved by Beijing Tsinghua Changgung Hospital.

## Author contributions

ZYW: Conceptualization, methodology, writing- original draft preparation. ZG: Software, data curation. XW: Methodology, visualization. HL: Methodology, investigation. FC: Formal analysis, writing- reviewing and editing. YL: Software. ZW: Supervision, project administration. All authors contributed to the article and approved the submitted version.

## References

[1] Saguil A Fargo MV. Acute respiratory distress syndrome: diagnosis and management. Am Fam Phys (2020) 101:730–38. Available at: https://www.aafp.org/pubs/afp/issues/2020/0615/p730.html. 32538594

[2] Vassiliou AG Kotanidou A Dimopoulou I Orfanos SE. Endothelial damage in acute respiratory distress syndrome. Int J Mol Sci (2020) 21(22):8793. doi: 10.3390/ijms21228793 33233715 PMC7699909

[3] Simmons S Erfinanda L Bartz C Kuebler WM. Novel mechanisms regulating endothelial barrier function in the pulmonary microcirculation. J Physiol (2019) 597:997–1021. doi: 10.1113/JP276245 30015354 PMC6375872

[4] Huppert LA Matthay MA Ware LB. Pathogenesis of acute respiratory distress syndrome. Semin Respir Crit Care Med (2019) 40:31–9. doi: 10.1055/s-0039-1683996 PMC7060969 31060086

[5] Qiu N Xu X He Y. LncRNA TUG1 alleviates sepsis-induced acute lung injury by targeting miR-34b-5p/GAB1. BMC Pulm Med (2020) 20:49. doi: 10.1186/s12890-020-1084-3 32087725 PMC7036216

[6] Shao L Meng D Yang F Song H Tang D. Irisin-mediated protective effect on LPS-induced acute lung injury *via* suppressing inflammation and apoptosis of alveolar epithelial cells. Biochem Biophys Res Commun (2017) 487:194–200. doi: 10.1016/j.bbrc.2017.04.020 28396150

[7] Gao X Huang C Geng T Chen X Wang J Liu J. Serum and urine metabolomics based on UPLC-Q-TOF/MS reveals the antipyretic mechanism of reduning injection in a rat model. J Ethnopharmacol (2020) 250:112429. doi: 10.1016/j.jep.2019.112429 31812644

[8] Ding Y Ma L He L Xu Q Zhang Z Zhang Z. A strategy for attenuation of acute radiation-induced lung injury using crocetin from gardenia fruit. BioMed Pharmacother (2022) 149:112899. doi: 10.1016/j.biopha.2022.112899 35366531

[9] Yeh YC Doan LH Huang ZY Chu LW Shi TH Lee YR. Honeysuckle (Lonicera japonica) and huangqi (Astragalus membranaceus) suppress SARS-CoV-2 entry and COVID-19 related cytokine storm *in vitro* . Front Pharmacol (2021) 12:765553. doi: 10.3389/fphar.2021.765553 35401158 PMC8990830

[10] Zhang J Li Y Wan J Zhang M Li C Lin J. Artesunate: a review of its therapeutic insights in respiratory diseases. Phytomedicine (2022) 104:154259. doi: 10.1016/j.phymed.2022.154259 35849970

[11] Zhang Q Yi H Yao H Lu L He G Wu M. Artemisinin derivatives inhibit non-small cell lung cancer cells through induction of ROS-dependent Apoptosis/Ferroptosis. J Cancer (2021) 12:4075–85. doi: 10.7150/jca.57054 PMC8176242 34093811

[12] Jia S Luo H Liu X Fan X Huang Z Lu S. Dissecting the novel mechanism of reduning injection in treating coronavirus disease 2019 (COVID-19) based on network pharmacology and experimental verification. J Ethnopharmacol (2021) 273:113871. doi: 10.1016/j.jep.2021.113871 33485971 PMC7825842

[13] Ma Q Xie Y Wang Z Lei B Chen R Liu B. Efficacy and safety of ReDuNing injection as a treatment for COVID-19 and its inhibitory effect against SARS-CoV-2. J Ethnopharmacol (2021) 279:114367. doi: 10.1016/j.jep.2021.114367 34174375 PMC8223030

[14] Xu X Zhang J Zheng W Yang Z Zhao X Wang C. Efficacy and safety of reduning injection in the treatment of COVID-19: a randomized, multicenter clinical study. Ann Palliat Med (2021) 10:5146–55. doi: 10.21037/apm-20-2121 33894725

[15] Tang LP Xiao W Li YF Li HB Wang ZZ Yao XS. Anti-inflammatory effects of reduning injection on lipopolysaccharide-induced acute lung injury of rats. Chin J Integr Med (2014) 20:591–99. doi: 10.1007/s11655-014-1758-x PMC7101712 24916807

[16] Yang C Song C Liu Y Qu J Li H Xiao W. Re-Du-Ning injection ameliorates LPS-induced lung injury through inhibiting neutrophil extracellular traps formation. Phytomedicine (2021) 90:153635. doi: 10.1016/j.phymed.2021.153635 34229173 PMC8213523

[17] Wang Z Wang X Guo Z Liao H Chai Y Wang Z. Reduning attenuates LPS-induced human unmilical vein endothelial cells (HUVECs) apoptosis through PI3K-AKT signaling pathway. Front Pharmacol (2022) 13:921337. doi: 10.3389/fphar.2022.921337 35903333 PMC9315302

[18] Wang Z Wang X Guo Z Liao H Chai Y Wang Z. In silico high-throughput screening system for AKT1 activators with therapeutic applications in sepsis acute lung injury. Front Cell Infect Microbiol (2022) 12:1050497. doi: 10.3389/fcimb.2022.1050497 36579349 PMC9792167

[19] Wang Z Chen W Li Y Zhang S Lou H Lu X. Reduning injection and its effective constituent luteoloside protect against sepsis partly *via* inhibition of HMGB1/TLR4/NF-kappaB/MAPKs signaling pathways. J Ethnopharmacol (2021) 270:113783. doi: 10.1016/j.jep.2021.113783 33421596

[20] Hu Q Hao C Tang S. From sepsis to acute respiratory distress syndrome (ARDS): emerging preventive strategies based on molecular and genetic researches. Biosci Rep (2020) 40(5):BSR20200830. doi: 10.1042/BSR20200830 32319516 PMC7199454

[21] Matthay MA Zemans RL Zimmerman GA Arabi YM Beitler JR Mercat A. Acute respiratory distress syndrome. Nat Rev Dis Primers (2019) 5:18. doi: 10.1038/s41572-019-0069-0 30872586 PMC6709677

[22] Wichterman KA Baue AE Chaudry IH. Sepsis and septic shock–a review of laboratory models and a proposal. J Surg Res (1980) 29:189–201. doi: 10.1016/0022-4804(80)90037-2 6997619

[23] Chang R Holcomb JB Johansson PI Pati S Schreiber MA Wade CE. Plasma resuscitation improved survival in a cecal ligation and puncture rat model of sepsis. Shock (2018) 49:53–61. doi: 10.1097/SHK.0000000000000918 28591008 PMC5718978

[24] Zhang H Liu J Zhou Y Qu M Wang Y Guo K. Neutrophil extracellular traps mediate m(6)A modification and regulates sepsis-associated acute lung injury by activating ferroptosis in alveolar epithelial cells. Int J Biol Sci (2022) 18:3337–57. doi: 10.7150/ijbs.69141 PMC9134924 35637949

[25] Wang JF Wang YP Xie J Zhao ZZ Gupta S Guo Y. Upregulated PD-L1 delays human neutrophil apoptosis and promotes lung injury in an experimental mouse model of sepsis. Blood (2021) 138:806–10. doi: 10.1182/blood.2020009417 34473230

[26] Olander A Bremer A Sundler AJ Hagiwara MA Andersson H. Assessment of patients with suspected sepsis in ambulance services: a qualitative interview study. BMC Emerg Med (2021) 21:45. doi: 10.1186/s12873-021-00440-4 33836665 PMC8033740

[27] Povoa P Coelho L. Which biomarkers can be used as diagnostic tools for infection in suspected sepsis? Semin Respir Crit Care Med (2021) 42:662–71. doi: 10.1055/s-0041-1735148 34544183

[28] Guo B Zhao C Zhang C Xiao Y Yan G Liu L. Elucidation of the anti-inflammatory mechanism of er miao San by integrative approach of network pharmacology and experimental verification. Pharmacol Res (2022) 175:106000. doi: 10.1016/j.phrs.2021.106000 34838694

[29] Huang YF Bai C He F Xie Y Zhou H. Review on the potential action mechanisms of Chinese medicines in treating coronavirus disease 2019 (COVID-19). Pharmacol Res (2020) 158:104939. doi: 10.1016/j.phrs.2020.104939 32445956 PMC7239792

[30] Gill SE Rohan M Mehta S. Role of pulmonary microvascular endothelial cell apoptosis in murine sepsis-induced lung injury *in vivo* . Respir Res (2015) 16:109. doi: 10.1186/s12931-015-0266-7 26376777 PMC4574190

[31] Ren Y Li L Wang MM Cao LP Sun ZR Yang ZZ. Pravastatin attenuates sepsis-induced acute lung injury through decreasing pulmonary microvascular permeability *via* inhibition of cav-1/eNOS pathway. Int Immunopharmacol (2021) 100:108077. doi: 10.1016/j.intimp.2021.108077 34464887

[32] Dai Y Liu J Zhang X Min X Wu J Du S. HSPA12A improves endothelial integrity to attenuate lung injury during endotoxemia through activating ERKs and akt-dependent signaling. Int Immunopharmacol (2021) 99:107987. doi: 10.1016/j.intimp.2021.107987 34343936

[33] Li F Li J Wang PH Yang N Huang J Ou J. SARS-CoV-2 spike promotes inflammation and apoptosis through autophagy by ROS-suppressed PI3K/AKT/mTOR signaling. Biochim Biophys Acta Mol Basis Dis (2021) 1867:166260. doi: 10.1016/j.bbadis.2021.166260 34461258 PMC8390448

[34] Mizuta Y Akahoshi T Guo J Zhang S Narahara S Kawano T. Exosomes from adipose tissue-derived mesenchymal stem cells ameliorate histone-induced acute lung injury by activating the PI3K/Akt pathway in endothelial cells. Stem Cell Res Ther (2020) 11:508. doi: 10.1186/s13287-020-02015-9 33246503 PMC7691956

[35] Bock FJ Tait S. Mitochondria as multifaceted regulators of cell death. Nat Rev Mol Cell Biol (2020) 21:85–100. doi: 10.1038/s41580-019-0173-8 31636403

[36] Abate M Festa A Falco M Lombardi A Luce A Grimaldi A. Mitochondria as playmakers of apoptosis, autophagy and senescence. Semin Cell Dev Biol (2020) 98:139–53. doi: 10.1016/j.semcdb.2019.05.022 31154010

