# Supplementary material for: Reduning alleviates sepsis-induced acute lung injury by reducing apoptosis of pulmonary microvascular endothelial cells
Source: Front Immunol. 2023 Jul 3;14:1196350. doi: 10.3389/fimmu.2023.1196350 (PMC10350519; doi:10.3389/fimmu.2023.1196350)
Supplement: Supplementary file 1 [file DataSheet_1.docx]

Supplementary Material

Reduning alleviates sepsis acute lung injury by reducing apoptosis of pulmonary microvascular endothelial cells (PMECs)

First Author*, Ziyi Wang

## * Correspondence: Zhong Wang: [wz523@mail.tsinghua.edu.cn](mailto:wz523@mail.tsinghua.edu.cn)

## Supplementary Figures


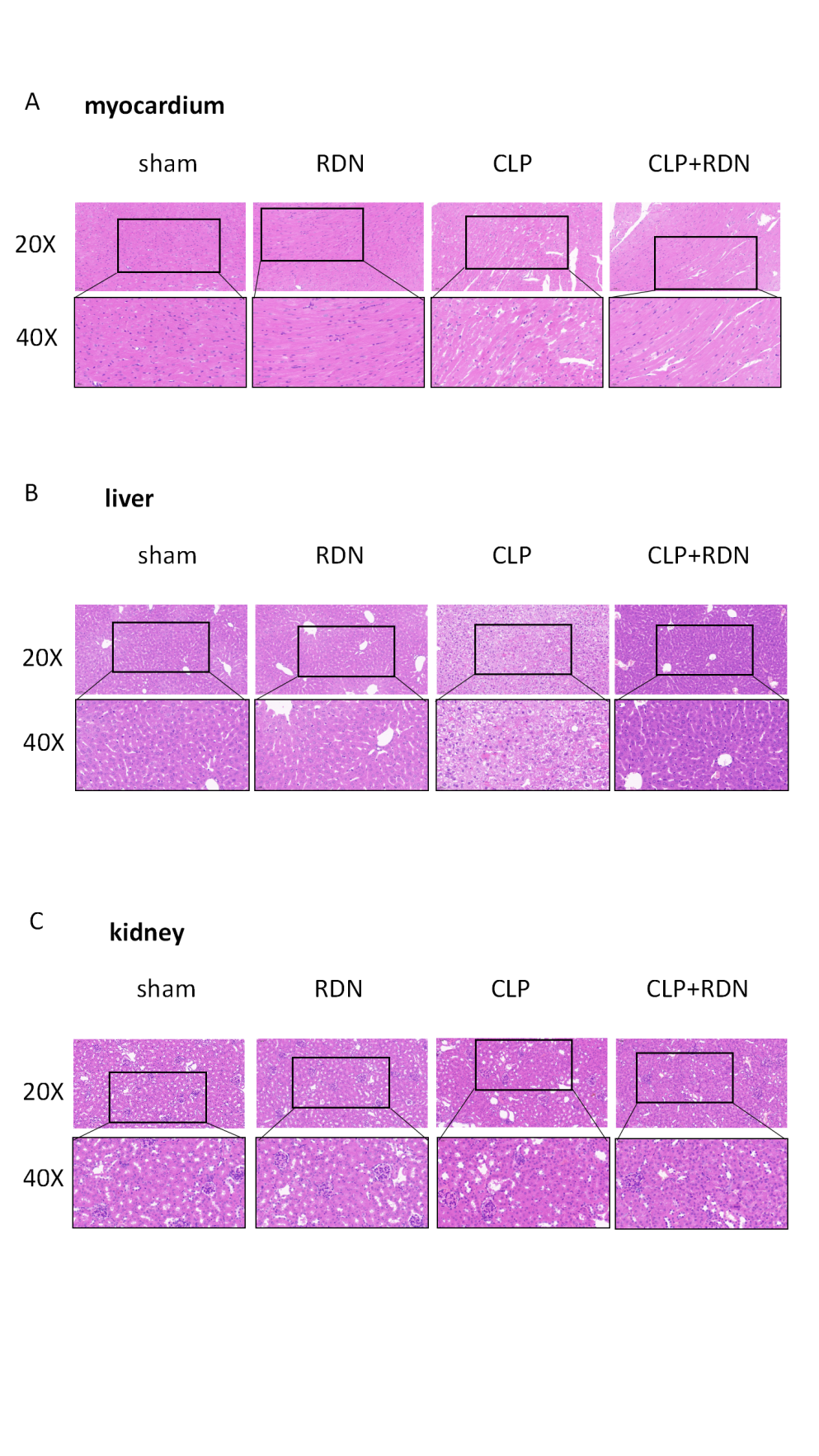


**Supplementary Figure 1.** (A)HE staining was used to examine myocardial structural changes in different groups of mice. The myocardial fibers of sham group and RDN group were arranged in a regular manner, the myocardial cells were eosinophilic staining, the transverse lines were clear, the cell membrane was intact, and the interstitial was not exudated. In CLP group, the myocardial fibers showed obvious wavy changes, some irregular arrangement, enhanced eosinophilic staining, blurred transverse lines, partially intact cell membranes, and exudation in the interstroma. Myocardial fibers in RDN group showed slight wavy changes, regular arrangement, and eosinophilic staining of cardiomyocytes with clear transverse lines, intact cell membranes, and slight exudation of interstroma. (B) HE staining was used to examine the changes of liver structure in different groups of mice. Compared with sham and RDN groups, the liver tissue of CLP group was infiltrated by inflammatory cells and liver tissue was significantly damaged, while in CLP+RDN group, the inflammatory cell infiltration was reduced and the liver tissue structure was relatively complete, similar to that of the control group. (C) HE staining was used to examine the changes of renal structure in different groups of mice. Compared with sham and RDN groups, renal tubules in CLP group showed obvious damage and increased inflammatory cell infiltration, while in CLP+RDN group, inflammatory cell infiltration was reduced and renal tubules were relatively intact, similar to the control group. Representative images of H&E-stained kidneys of the three groups at 20X (upper panels) and 40X (low panels) magnification; scale bars are shown in figures.
